# The omega-3 and Nano-curcumin effects on vascular cell adhesion molecule (VCAM) in episodic migraine patients: a randomized clinical trial

**DOI:** 10.1186/s13104-021-05700-x

**Published:** 2021-07-23

**Authors:** Mina Abdolahi, Elmira Karimi, Payam Sarraf, Abbas Tafakhori, Goli Siri, Farahnaz Salehinia, Mohsen Sedighiyan, Behzad Asanjarani, Mostafa Badeli, Hamed Abdollahi, Niyoosha Yoosefi, Abolghasem yousefi, Amir Shayegan rad, Mahmoud Djalali

**Affiliations:** 1 Amir Alam Hospital Complexes, Tehran University of Medical Sciences, Sa’adi Street, Tehran, Iran; 2 Department of Community Nutrition, School of Nutritional Sciences and Dietetics, Tehran University of Medical Sciences, Poursina Street, Tehran, Iran; 3 Iranian Centre of Neurological Research, Neuroscience Institute, Tehran University of Medical Sciences, Tehran, Iran; 4 Imam Khomeini Hospital, Tehran University of Medical Sciences, Tehran, Iran; 5 Department of Internal Medicine, Amiralam Hospital, Tehran University of Medical Sciences, Tehran, Iran; 6 Department of Cellular and Molecular Nutrition, School of Nutritional Sciences and Dietetics, Tehran University of Medical Sciences, Poursina Street, PO Box: 14155-6446, Tehran, Iran; 7 Department of Nutrition, School of Nutritional Sciences and Dietetics, Tehran University of Medical Sciences, Poursina Street, Tehran, Iran; 8 Department of Anesthesiology, Amir Alam Hospital Complexes, Tehran University of Medical Sciences, Sa’adi Street, Tehran, Iran; 9 Honours Cellular Anatomical Physiology, University of British Columbia, Vancouver, BC Canada

**Keywords:** Migraine, Vascular cell adhesion molecule, Gene expression, Serum concentration, Omega-3, Nanocurcumin

## Abstract

**Objective:**

The purpose of this clinical trial was to examine the effect of omega-3 fatty acids (W-3 FAs), nanocurcumin and their combination on serum levels and gene expression of VCAM in patients with episodic migraine.

**Results:**

In this study, 80 patients were randomly divided in to 4 groups to receive for 2 months. Both serum levels and gene expression of VCAM showed remarkable decreases after single W-3 and after combined W-3 and nanocurcumin interventions. However, a borderline significant change and no remarkable change were observed after single nanocurcumin supplementation and in control group, respectively. While a significant difference between study groups in VCAM concentrations existed, there was no meaningful difference in VCAM gene expression among groups. It appears that the W-3 and combined W-3 and nanocurcumin can relieve VCAM serum level and its gene expression in patients with episodic migraine. Moreover, the combination of W-3 with nanocurcumin might cause more significant declines in VCAM level in the serum of migraine patients than when W-3 is administered alone.

*Trial Registration*: This study was registered in Iranian Registry of Clinical Trials (IRCT) with ID number: NCT02532023.

**Supplementary Information:**

The online version contains supplementary material available at 10.1186/s13104-021-05700-x.

## Introduction

Migraine is a neurovascular disturbance characterized by throbbing, unilateral headaches accompanied by photophobia, intolerance of light and autonomic features [1]. Neurogenic inflammation enhances sensitivity of the as nociceptors [2] which up-regulate the secretion of pro-inflammatory factors [3, 4]. These cytokines trigger the permeability of vascular system, suggesting a mechanistic role of them in elevating the expression of endothelium-derived products such as VCAM, ICAM and selections resulted in headaches severity [5, 6].

Based on evidences the proinflammatory cytokines such as TNF-α are vasodilators and induce VCAM expression. These are associated with microglia activation which causes brain inflammation and neuropathic pain [5, 6]. Several medications used to treat migraines include NSAIDs, tricyclic antidepressants such as amitriptyline, COX-2 inhibitors, and sodium valproate [7]. The pain-alleviating power of most of migraine drugs is due to their anti-inflammatory effect and preventing from expression of endothelium-derived factors [8–14]. Valproate sodium inhibits mRNA expression of endothelial inflammation mediators including TNF-α, IL-1β and IL-6 as well as T and B cells accumulation in macrophages [8, 9]. It also inhibits NF-κB/iNOS signaling resulted in reducing endothelial inflammation [10]. NSAIDs and Cox-2 inhibitors suppress activated endothelial and pain by inhibiting cyclooxygenase enzyme and gene expression of inflammatory endothelial factors (VEGF) [11, 12]. Amitriptyline that widely used in the treatment of migraine, can inhibit the ICAM-1, VCAM-1, iNOS, and COX-2 expression lead to inhibiting endothelial-derived inflammation [14].

Existing evidences have shown that W-3 and curcumin have the same anti-inflammatory effects as the drugs in migraine treatment without any serious side effect [15, 16].

Curcumin have no significant side effects, even in doses higher than 10 g [17]. The nano-curcumin increases the absorption of curcumin up to 40-fold in rats and 27-fold in humans and [18] is safe up to a dose of 210 mg [19]. A meta-analysis showed that curcumin up to 6 g is completely safe [20]. In the case of W-3, a meta-analysis demonstrated that consumption omega-3 (DHA + EPA) up to 4 gr is safe and has no significant side effect [21]. Additionally, in previous clinical trials, a combination of 2.5 gr of omega-3 and 80 mg of nanocurcumin was well tolerated and had no side effects [22, 23].

There are promising findings regarding the efficacy of W-3 and curcumin on VCAM reduction [24–27]. Moreover, some scientific documents have reported the elevated anti-inflammatory activity of lower dosages of W-3 and curcumin, when prescribed in combination [22, 23, 28–31]. This study aimed to examine the efficacy of supplementation with W-3, nanocurcumin and their combination on serum concentrations and mRNA expression of VCAM in patients with episodic migraine.

## Main text

### Method and materials

#### Design and population

Among 285 subjects who attended to Sina hospital neurology clinic in Tehran, 80 men and women were recruited to this double-blind (neither the participants nor the experimenters) randomized controlled trial on July 2015. Participants were adults and had a current diagnosis of episodic migraine in accordance with the IHS criteria (≥ 15 headache days per month for more than 3 months or ≥ 1 attack per week) [32]. All recruiting criteria are shown in Table 1.

**Table 1:** Inclusion and exclusion criteria

People were considered as eligible if they meet all of the following criteria	People did not enter the trial if they meet one or more of the following criteria	Participants were excluded during the trial if they meet one or more of the following criteria
BMI greater than or equal to 18.5 kg/m2	A history or current diagnosis of kidney, liver or thyroid disorders, heart diseases, diabetes, different malignancies and other inflammatory comorbidities	Allergic reaction to W-3 or nanocurcumin supplements
Males and females 20–50 years of age	Low BMI (less than 18.5 kg/m2)	Lack of patient's cooperation
Episodic migraine	Dietary supplementation during the preceding three months	Pregnancy during the study period
Stable diet, physical activity or supplements during the trial	Pregnancy, lactation	Self-reported changes in diet and lifestyle
willingness to participate	Menopause	Changes in dosage or type of routine medications
	Alcohol or drug abuse and cigarette smoking during the preceding six months	Over 2 weeks anti-inflammatory drugs consumption
		Compliance rate less than 90%

#### Omega-3 and nanocurcumin supplementation

The stratified randomization method based on sex and BMI was used to assign participants in 4 groups to receive 2 months supplementation of Group 1) 2 capsules containing 600 mg EPA + 300 mg DHA + 100 mg other W-3 + 1 nanocurcumin placebo, Group 2) 1 capsule containing 80 mg nanocurcumin + 2 W-3 placebo, 3) 2 capsules containing 600 mg EPA + 300 mg DHA + 100 mg other W-3 + 1 capsule containing 80 mg nanocurcumin (group 3) and 4) 2 W-3 placebo + 1 nanocurcumin placebo (control group).

Omega 3 supplement (Omega MAX) and its placebo that were completely similar in shape and color were provided by Zahravi Pharmaceutical Company. Each 1250 mg capsule of W-3 contains 600 mg of EPA, 300 mg of DHA, 100 mg of other W-3 FAs, gelatin, glycerin and pure water. Nanocurcumin supplement (Sinacurcumin 80) and its placebo, which were completely similar in shape and color, were prepared by Sinacurcumin Pharmaceutical Company. Each 80 mg nanocurcumin capsule contains approximately 100% curcumin along with much lower amounts of other compounds such as curcuminoid, emulsifier (polysorbate), gelatin and glycerin. Both omega-3 and nano-curcumin placebo were made from oral paraffin.

Random allocation and enrolling participants to the study were done by trained people who were outside the investigation. Administration of 25–50 mg three cyclic antidepressants (amitriptyline or nortriptyline) and 20–40 mg of β-blockers (eg propranolol) along with study supplements was given to all groups. Patients were asked to abstain from altering their dietary and physical activity during the intervention. We instructed patients to consume NSAIDS in times of severe headache attacks. To control the bias of NSAID consumption, patients were trained that taking of these drugs should not exceed two weeks during the two months of study and if they take more than this amount, they will be excluded from the study. Patients were asked to record the type and number of medications taken. In our study, none of the patients had used analgesia for more than a week during the study.

#### Questionnaires

A predesigned questionnaire was used to consider general characteristics of migraine patients including gender, age, financial perception, migraine duration, educational status, history of other diseases and use of medications and dietary supplements. Severity, numbers and duration of headaches were examined by a trained clinician. Scales for headache severity was within 1–10 in which score 1 and 10 were indicative of minimum and maximum pain of headaches.

#### Anthropometric indices

Measured anthropometric indices included: i) weight (through 803, Seca Clara, Germany with 0.1 kg accuracy with light clothing and no shoes); ii) height (by a stadiometer (Seca) with 0.1 cm accuracy); and iii) WC (with a common tape after normal exhalation).

### Statistical analysis

Sample size was computed based on the VCAM-1 variable considering the difference of 1.817 ng/dl between the intervention and control groups via the following formula (If α = 0.05, 1 − β = 1.28 (Power = 90%)) [33].

$${\text{n}} = \left( {\frac{{{\text{Z}}\,1 - \alpha /2 + {\text{Z}}\,1 - \beta }}{{\text{d}}}} \right)^{2}$$

N = 17 was obtained for each study group. By considering 20% missing during intervention, 20 subjects were selected for each study groups. Data normality was checked through Kolmogorov–Smirnov distribution. For comparison of normal quantitative data means among groups and in each group, ANOVA paired t-tests were conducted, respectively. We used kruskal–wallis and Wilcoxon to compare abnormal quantitative variables between groups and in each single group, respectively. We conducted ANCOVA test to compare study outcomes between groups with adjustment of confounding factors (BMI, energy intake and age). The data were finally analyzed by SPSS version 22, in which P-value ≤ 0.05 was considered as significant.

### Measurement of serum concentration and gene expression of VCAM

Laboratory measurements were done at Tehran University of Medical Sciences. At baseline and after 2-month intervention, two blood samples from subjects were collected including 10 ml in tubes containing heparin for PBMCs separation and 5 ml for serum separation. Total RNA from cells were obtained using RNeasy Plus Mini Kit (Qiagen, Valencia, CA, USA). The purified RNA was reversed to synthesis of DNA using QuantiTect Reverse Transcription Kit (Qiagen, Germany). In the PCR step, appropriate primer for VCAM was designed using Primer Express 3 software (Applied Biosystems). Whole genome sequences of VCAM primer were forward: 5'-TAGCGTGTACCCCCTTGACC-3' and reverse: 3'-AACTTAGCCTGACAAACAAGAGC-5'. PCR Analysis were performed by StepOne system (Applied Biosystems, Foster City, CA, USA) using 7 μL of Enzymes (Taq polymerase, SYBR Green, etc.), 2 μL of cDNA, 0.5 μL of each forward and reverse primers in an ultimate volume of 20 μL [34]. VCAM expression calculation was conducted by Ct (2-ΔΔCt) equation. Blood serums were stored in -80 °C until serum VCAM analysis via ELISA method (Bioassay Technology Laboratory, Chain).

## Results

### Basic patient information

From the 80 individuals (20 participants in each group), 6 subjects (1 woman in group 1, 1 woman in group 2 and 1 woman and 2 mans in group 3 and 1 woman in group 4) did not continue with the trial because of changing their treatment trends or drugs (Additional file 1: Figure S1). No serious harms or unintended effects were reported from study supplements and compliance rate of the patients was 100%. A summary of baseline characteristics of participants is demonstrated in Table 2. No significant between-group differences in general, biochemical and clinical characteristics was detected (P > 0.05).

**Table 2:** Baseline characteristic of participants

Characteristic	Groups				P-value
	Group 1 (N = 19)	Group 2 (N = 19)	Group 3 (N = 17)	Group 4 (N = 19)	
Gender (female/male)	(16/4)	(16/4)	(16/4)	(16/4)	
Age	36.20 ± 1.89	37.10 ± 1.78	36.90 ± 1.84	36.45 ± 1.87	0.97
Weight (kg)	69.45 ± 2.75	71.65 ± 3.80	69.25 ± 2.51	70.95 ± 2.31	0.92
Height (cm)	162.70 ± 1.92	161.25 ± 1.76	162.80 ± 1.97	162.20 ± 1.52	0.92
WC (cm)	79.80 ± 1.57	83.30 ± 2.20	80.60 ± 1.67	83.20 ± 1.65	0.39
BMI (kg/m^2^)	26.23 ± 0.94	27.29 ± 1.04	26.17 ± 0.84	27.13 ± 0.87	0.75
EI(kcal)	1612.12 ± 173.04	1703.79 ± 215.20	1592.36 ± 105.53	1410.69 ± 68.15	0.11
VCAM gene expression	15.67 ± 0.53	15.74 ± 0.74	15.55 ± 0.89	14.58 ± 0.69	0.92
Serum VCAM concentration (ng/dl)	4.90 ± 1.15	4.18 ± 0.72	4.75 ± 1.06	4.94 ± 1.22	0.99
Headache attacks (number per week)	2.81 ± 0.31	2.77 ± 0.26	2.72 ± 0.40	2.76 ± 0.24	0.99
Headache severity (scoring from 0–10)	7.52 ± 0.44	7.36 ± 0.46	7.47 ± 0.48	7.39 ± 0.46	0.99
Headache duration (hours)	7.44 ± 1.80	7.68 ± 1.77	7.28 ± 1.77	7.34 ± 1.43	0.99

ANOVA test; *BMI* Body mass index, *EI* Energy intake. Data are presented as mean ± SE (standard error)

### Outcome assessment

Table 3 summarizes the results of the study. Two-month supplementation in group 1 and 3 caused statistically significant reductions of both expression and serum levels of VCAM in the participants. Additionally, there were near to significant changes in VCAM serum levels and gene expression in group 2. No meaningful change in study outcomes was observed in control group. Moreover, significant difference was presented in VCAM serum concentrations among all groups. Although, VCAM expression difference between 4 groups was not significant.

**Table 3:** Comparison of study outcomes between study groups before and after supplementation in each group

Parameters	Time	Groups				P-value
		Group 1 (N = 19)	Group 2 (N = 19)	Group 3 (N = 17)	Group 4 (N = 19)	
VCAM gene expression	Before	15.67 ± 0.53	15.74 ± 0.74	15.55 ± 0.89	14.58 ± 0.69	0.92
	After	13.99 ± 0.95	14.09 ± 0.87	13.34 ± 0.90	14.02 ± 0.61	0.63
	Change	− 1.68 ± 0.82	− 1.64 ± 0.85	− 2.21 ± 0.56	0.55 ± 0.61	0.46
	P-value	0.05	0.06	0.001	0.37	
Serum VCAM concentration (ng/dl)	Before	4.90 ± 1.15	4.18 ± 0.72	4.75 ± 1.06	4.94 ± 1.22	0.99
	After	4.40 ± 1.11	3.88 ± 0.72	3.50 ± 0.74	4.84 ± 1.21	0.94
	Change	− 0.50 ± 0.34	− 0.30 ± 0.21	− 1.24 ± 0.42	− 0.09 ± 0.18	0.05
	P	0.02	0.06	0.004	0.19	
Headache attacks (number per week)	Before	2.81 ± 0.31	2.77 ± 0.26	2.72 ± 0.40	2.76 ± 0.24	0.99
	After	1.82 ± 0.27	1.67 ± 0.42	0.62 ± 0.08	2.22 ± 0.38	0.009
	Change	− 0.98 ± 0.33	− 1.10 ± 0.33	− 2.09 ± 0.34	− 53 ± 0.32	0.01
	P-value	0.009	0.004	< 0.001	0.11	
Headache severity (scoring from 0–10)	Before	7.52 ± 0.44	7.36 ± 0.46	7.47 ± 0.48	7.39 ± 0.46	0.99
	After	5.86 ± 0.48	5.42 ± 0.52	5.02 ± 0.56	6.78 ± 0.43	0.02
	Change	− 1.65 ± 0.44	− 1.94 ± 0.49	− 2.44 ± 0.41	− 0.60 ± 0.48	0.04
	P-value	0.001	0.001	< 0.001	0.22	
Headache duration (hours)	Before	7.44 ± 1.80	7.68 ± 1.77	7.28 ± 1.77	7.34 ± 1.43	0.99
	After	6.02 ± 1.36	5.26 ± 1.66	4.15 ± 1.47	6.68 ± 1.13	0.64
	Change	− 1.42 ± 0.96	− 2.42 ± 0.67	− 3.12 ± 0.96	0.65 ± 1.03	0.26
	P-value	0.15	0.002	0.005	0.53	

*VCAM* Vascular cell adhesion molecule

^***^AVCOVA test (adjusted for BMI, age and energy intake)

**Kruskal–Wallis test

*Wilcoxon test (Two related sample)

## Discussion

Our study for the first time, evaluates whether W-3, nanocurcumin and their combination could reduce serum levels and mRNA expression of VCAM in episodic migraine. This research showed that single W-3 and accompanied W-3 and nanocurcumin supplementation for 2 months led to significant decreases in gene expression and blood levels of VCAM in participants. As we expected, the change of both outcomes in group 3 was greater than group 1. The changes of VCAM serum concentrations was significant within 4 groups. However, VCAM expression was not statistically significant among 4 groups of trial, perhaps due to small sample size. Previously, we found treatment with combined W-3 and nanocurcumin caused meaningful reductions in severity and frequency of migraine headaches [22]. We suspect greater reductions in serum concentrations when accompanied W-3 and nanocurcumin are administered in migraine patients might be one of the underlying mechanisms for the significant alleviation in headaches severity and frequency in comparison to when they are administered alone might. The findings of the current intervention contradict with Poreba et al.’s study where W-3 had no effect on serum VCAM in obese population [35]. A meta-analysis reported no meaningful effect of W-3 on serum VCAM [36]. These results could be due to differences in study duration, population and W-3 dosage. To date, there is no study on the investigation of accompanied W-3 and nanocurcumin supplementation on endothelial factors in human. Our findings were inconsistent with Liu et al.’s study in mice that showed the significant decreasing effect of curcumin supplementation on VCAM expression (37). This controversy could be due to differences between human and animal studies. Experimental studies have demonstrated W-3 suppress VCAM-1 expression by inhibition of PPAR-γ [38], IRF-1 and miR-126 expression (39), sirtuin-1 production [40] and nuclear factor of kappa light polypeptide gene enhancer in B-cells inhibitor, alpha (IκBα) and NFKB phosphorylation [41] which are involved in vascular adhesion molecules formation. These FAs also inhibit macrophage activity and decrease the VCAM-1 levels resulted in attenuating severity of migraine headaches [42].

The next mechanism of W-3 against VCAM elevation is up-regulating influence of W-3 on the TREK1 potassium channels as maintainers of the blood–brain barrier integrity which has been to block VCAM transition from the vessel [43]. Curcumin may reduce VCAM levels by down-regulating the phosphorylation of PI3-kinase/Akt, p38 MAPK and JNK [44] and NFKB expression (45), the migration and proliferation T-cells [37, 46], induction of anti-oxidative enzymes [47] and reduction of the expression and transcriptional activity of histone acetyltransferases including AP-1 and p300 which can effectively prevent VCAM production [48, 49].

Based on the results of current and previous studies, we suspect more reduction in VCAM serum level might be one of the mechanisms underlying the greater alleviations in severity and frequency of headaches observed in episodic migraine patients after combined W-3 and nanocurcumin interventions than single W-3 or nanocurcumin supplementations. Further researches are needed to confirm the evidence of the current investigation.

## Limitations

There were several limitations including small sample size and short duration of study. Additionally, the mechanistic explanations in the experimental studies mentioned in this study should be attributed to human with caution due to the different conditions exist in cell cultures and animal models [35].

## Supplementary Information

**Additional file 1**: **Figure S1**. Flow chart for the clinical trial. This Flow chart shows the number of participants in the four study groups, the number of missing in every group as well as participants entered to the analysis.

## Data Availability

The datasets supporting this article results are included within the manuscript. More information could be available by contact with this email address: Elmira.karimii1994@gmail.com.

## Abbreviations

MIDs: Mitochondrial disorders
VNs: Vasoactive neuropeptides
VCAM: Vascular cell adhesion molecule
ICAM: Intercellular adhesion molecule
NSAIDs: Non-steroidal anti-inflammatory drugs
W-3 FAs: Omega-3 fatty acids
NFkB: Nuclear factor-κB
HIS: Internatioanl Headache Society
PCR: Polymerase chain reaction
BMI: Body mass index
EPA: Eicosapentaenoic acid
DHA: Docosahexaenoic acid
PBMCs: Peripheral blood mononuclear cells
WC: Waist circumference
SPSS: Statistical Package for Social Sciences
ANOVA: Analysis of variance
ANCOVA: Analysis of covariance
IRF-1: Interferon regulatory factor
PPAR-γ: Peroxisome proliferator-activated receptor gamma
miR-126: MicroRNA 126
IκBα: Nuclear factor of kappa light polypeptide gene enhancer in B-cells inhibitor, alpha
PI3-kinase: Phosphoinositide 3-kinases
Akt or PKB: Protein kinase B
p38 MAPK: P38 MAP Kinase
JNK: C-Jun N-terminal kinases
AP-1: Activator protein 1
