# Supplementary figures and images for: The omega-3 and Nano-curcumin effects on vascular cell adhesion molecule (VCAM) in episodic migraine patients: a randomized clinical trial
Source: BMC Res Notes. 2021 Jul 23;14:283. doi: 10.1186/s13104-021-05700-x (PMC8305494; doi:10.1186/s13104-021-05700-x)

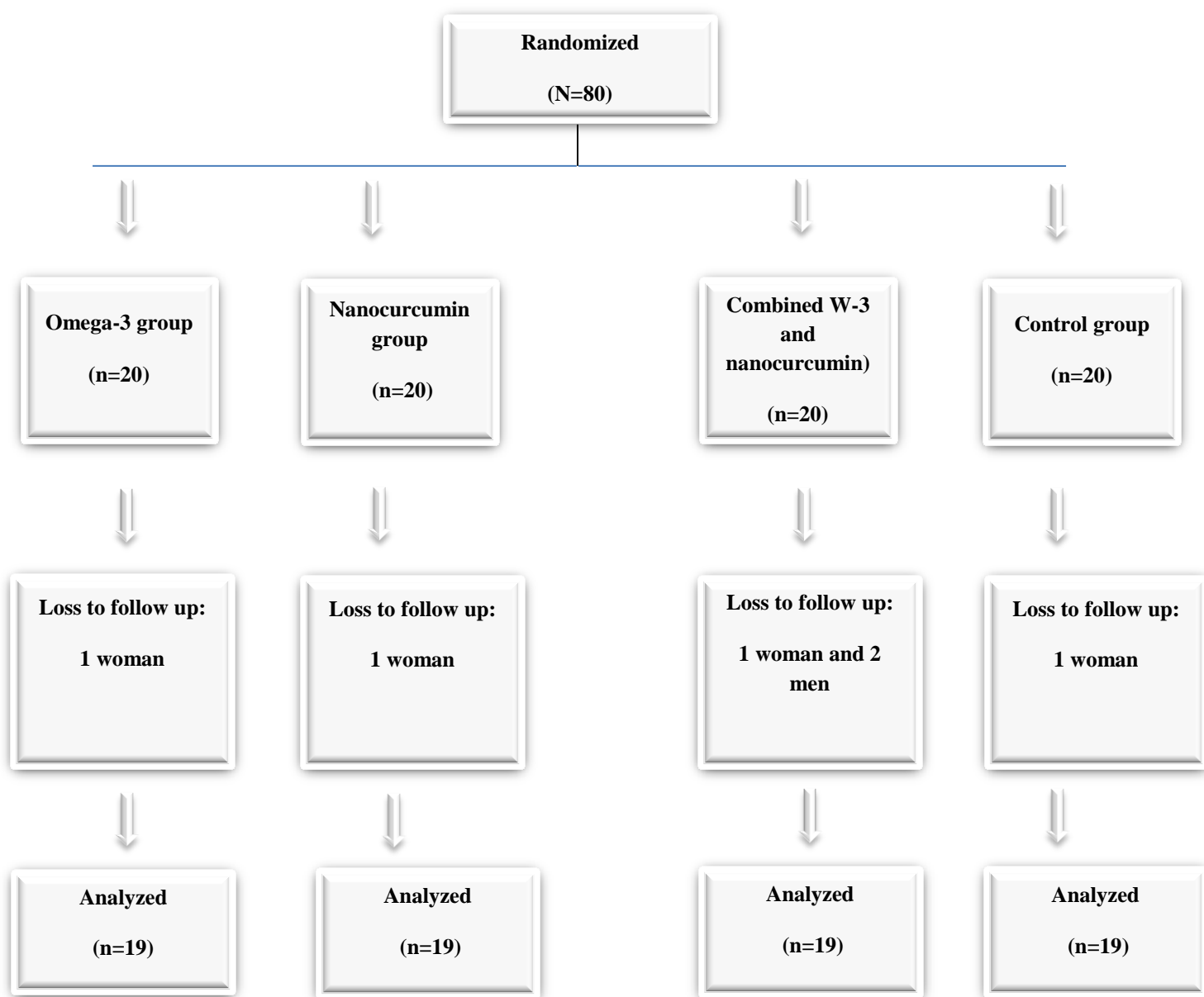

**Figure 1.** Flow chart for the trial

Supplement: Supplementary file 1 — Additional file 1: Figure S1. Flow chart for the clinical trial. This Flow chart shows the number of participants in the four study groups, the number of missing in every group as well as participants entered to the analysis. [file 13104_2021_5700_MOESM1_ESM.pdf]
